# The Ideal Operating-Theatre

**Published:** 1912-03-02

**Authors:** 


### THE IDEAL OPERATING-THEATRE.

In these days when one of the most widely discussed questions of hospital construction is the planning and equipment of the operating theatre, and when every surgeon or architect has his own ideas on the subject—often, as we have reason to know, very bizarre and extravagant ideas, too—it is interesting to read a common-sense discussion of the whole problem by an expert. Such a discussion appears in a recent number of the *Zeitschrift für Krankenpflege*, where Dr. Adler describes the essentials of the modern operating-room. The minimum essentials are simplicity, efficient ventilation, a maximum of light, roominess, and adaptability. Extravagantly designed and elaborately fitted theatres, such as those which were fitted up more than ten years ago in Breslau and which obtained a certain popularity in American hospitals, are open to very many objections. They are difficult to keep clean, and notwithstanding the superfluity of care taken to ensure that they shall be absolutely aseptic, the results obtained by work in them do not compare favourably with those given by smaller and less ambitious rooms where ordinary aseptic precautions are rigidly observed. One of the most frequent errors to be seen in the modern theatre which has been planned on the "faddist" principle is the complicated piece of apparatus that does duty as an operating table. Stability, in such cases, is obtained by the use of a ponderous and heavy central column, and ingenious mechanical contrivances are designed to raise and lower the heavy table itself. In one model an oil suction-pump is used; in another an electric raising-apparatus is substituted. These intricate and unduly complex labour-saving devices are so many additional pitfalls to the surgeon who has to use the table. A plain, simple design, which can be altered to secure the three positions most often required in general surgery—the Trendelenburg, lithotomy, and low-head position—fulfils all the requirements needed, and so long as it also secures stability, rigidity, and is marked by an absence of screws and bolts it is a useful and excellent operating-table; the surgeon who wants more should be required to provide his own apparatus and to pay for it. Already some of our hospitals possess operating theatres which answer every purpose but the one which their imaginative designers had in view. In London alone there are several such follies, and a tour through our provincial hospitals and infirmaries will largely add to their number. When the Breslau example was a law unto every progressive surgeon, theatre planning threatened to become as weird a process of construction as that which the architects of the mathematical king, in Mr. Stockton's entertaining fable, indulged in. Nowadays we are returning to the more sober stage when essentials and not theories are all important. Dr. Adler thinks the theatres at Pankow Hospital, and at the Bethanian Hospital at Berlin are as near perfection as it is possible to attain: we ourselves prefer the theatre at the new surgical clinic at Gottingen to either of these two. In England excellent theatres are to be found at the Leeds Infirmary, at St. Thomas's Hospital, and at many of our smaller institutions—for instance, at the Tottenham Hospital and at the Bedford Hospital. These all stand out by reason of their simplicity and adaptability; they are well lighted and well ventilated, and although they may lack the imposing appearance presented by the marble operating-theatres of such hospitals as Mount Sinai at New York or the Moscow Gynæcological Hospital, they are in every way ideal theatres under the conditions prevailing at their respective institutions. The real usefulness of an operating theatre is measured in results, and we think that a statistical enumeration of results from the various types of theatre will show that the simple model is eminently preferable to the more elaborate and complicated type. On economical grounds there is every reason to prefer the former, and from a structural and architectural, as well as from an administrative point of view, the simpler the theatre is the closer does it approach to the ideal.

